# Traveling Hammocks for Invalids

**Published:** 1875-07

**Authors:** 


Traveling Hammocks for Invalids.—A novel method for the conveyance of invalids by railway was tried last week with very great success. The patient, who had been treated in the Westminster Hospital for a severe burn, was conveyed by rail from the Victoria Station to Margate in one of Seydel's hammocks. The hammock was slung in a parcel van, (baggage car) which, on application, had been considerately lent by the managers of the London, Chatham & Dover Railway; and although the journey occupied nearly three hours, it proved perfectly painless, the patient experiencing neither jar nor jolt in the transit. This hammock-traveling possess so many advantages for invalids that its further development may be fairly expected. The railway companies would find it to their interest to provide the necessary accommodation for slinging; and there would be many worse methods of accomplishing a long night journey to Scotland, for instance, both for healthy persons and invalids, than swinging comfortably in a hammock, exempt from all the jerks and twists and jolts inseparable from a journey by railway at present express pace.—*Medical Times and Gazette, May* 29.

Why is a physician better taken care of than his patients? Because, when he goes to bed, somebody is sure to rap him up.

